# Increasing rate of inflammatory bowel disease: a 12-year retrospective study in NingXia, China

**DOI:** 10.1186/s12876-015-0405-0

**Published:** 2016-01-12

**Authors:** Huihong Zhai, Aiqin Liu, Wenyu Huang, Xin Liu, Shanshan Feng, Jing Wu, Yuping Yao, Chao Wang, Qianqian Li, Qian Hao, Jianguo Hu, Shutian Zhang

**Affiliations:** Department of Digestive Diseases, General Hospital of Ningxia Medical University, Yinchuan, 750004 China; Deparment of Digestive Diseases, Graduate School of Ningxia Medical University, Yinchuan, Ningxia 750004 China; Department of Gastroenterology, Beijing Friendship Hospital Affiliated to Capital Medical University, Beijing, 100050 China; Beijing Key Laboratory for Precancerous Lesion of Digestive Diseases, Beijing, 100050 China; National Clinical Research Center for Digestive Diseases, Yongan Street, Xicheng Area, Beijing, 100050 PR China

**Keywords:** Inflammatory bowel disease, NingXia, China, Retrospective analysis

## Abstract

**Background:**

In China, the incidence of Inflammatory bowel disease (IBD) has shown a significant growth trend. Analysis of the epidemiology, clinical manifestations, diagnostic means, and treatment of IBD will further improve the clinician's understanding of IBD, improve knowledge and further enable early diagnosis and standardized therapeutic management. The purpose of this study was to analyze the clinical characteristics of IBD inpatients in General Hospital of NingXia Medical University over a 12-year period to identify trends in clinical and epidemiological features, clinical manifestations, and treatment programs.

**Methods:**

By excluding188 patients with incomplete information or incompatible with the 2012 Guidlines cases, we retrospectively analyzed the case records of 567 inpatients with a diagnosis of IBD admitted to the General Hospital of NingXia Medical University between January 2002 and December 2014. The clinical epidemiological features, clinical manifestations, diagnostic methods, and therapeutic status were analyzed.

**Results:**

Over the study period, IBD hospitalization rates in 2002 and 2014 groups was 1.96 % and 4.05 %, increased 2.07 times. Of 567 cases of IBD, 483 (85.19 %) cases were categorized as ulcerative colitis (UC) and 84 as Crohn’s disease (CD) (14.81 %). Total male cases were 321 (56.61 %). Mean age of cases was 49.06 ± 14.92 years for UC and 44.84 ± 14.67 years for CD. The majority of UC was located in the colon, with a moderate level of disease activity. A combination of clinical manifestations and colonoscopy was mostly used to make a diagnosis; relatively the rate of pathological diagnosis was low, with a small proportion of patient’s diagnosed based on radiology. Treatment with SASP/5ASA and steroids was applied to the majority of inpatients and 47.83 % were treated with antibiotics; in contrast, only 1.86 % cases were treated with immunosuppressive therapy.

**Conclusions:**

An increasing trend of admissions for IBD can be observed in our study; there are some differences in clinical features and treatment compared with Western countries, and further research into this is required.

## Background

Inflammatory bowel disease (IBD) is a general diagnostic term that describes conditions with idiopathic, chronic, relapsing, and remitting inflammation of the gastrointestinal tract; include Ulcerative Colitis (UC) and Crohn’s Disease (CD), which has both overlapping and distinct clinical and pathological features. Although incidence and prevalence of IBD are beginning to stabilize in high-incidence areas, they continue to rise in low-incidence areas such as southern Europe, Asia, and much of the developing world [1, 2]. Current published data on IBD in Europe gives a prevalence of IBD as between 0.5 % and 1 % of the population. In China, the incidence has shown a significant growth trend [3–6] since 1956, when the Peking Union Medical College Hospital reported the first cases of IBD [7]. Despite this, the pathogenesis of IBD is incompletely understood. Genetic and environmental factors, such as altered luminal bacteria and enhanced intestinal permeability, play a role in the deregulation of intestinal immunity, leading to gastrointestinal injury. The clinical features of IBD may provide some clues to the cause of disease. IBD not only causes growth failure and retarded sexual development in young people, but can also result in psychological problems. Medical treatments such as corticosteroids or immunosuppressive drugs cause secondary health problems, and surgery may result in complications such as intestinal failure, colorectal cancer, or sepsis [8]. Analysis of the epidemiology, clinical manifestations, diagnostic means, and treatment of IBD will further improve the clinician's understanding of IBD, improve knowledge and further enable early diagnosis and standardized therapeutic management. To this end, the Chinese Medical Association of Gastroenterology has developed a research-based consensus opinion on the diagnosis and treatment of IBD, published and updated in 1978, 1993, 2000, 2007, and 2012. These revisions are dependent on the analysis of clinical data from Western countries due to a lack of large-scale epidemiological, clinical studies and an inadequate follow-up system in China. However, owing to differences in environment, ethnicity, lifestyle, and so on, disease characteristics will not be exactly the same. China is in urgent need of more multi-center, well-designed clinical research in order to develop treatment guidelines based on local conditions.

**Fig. 1:**
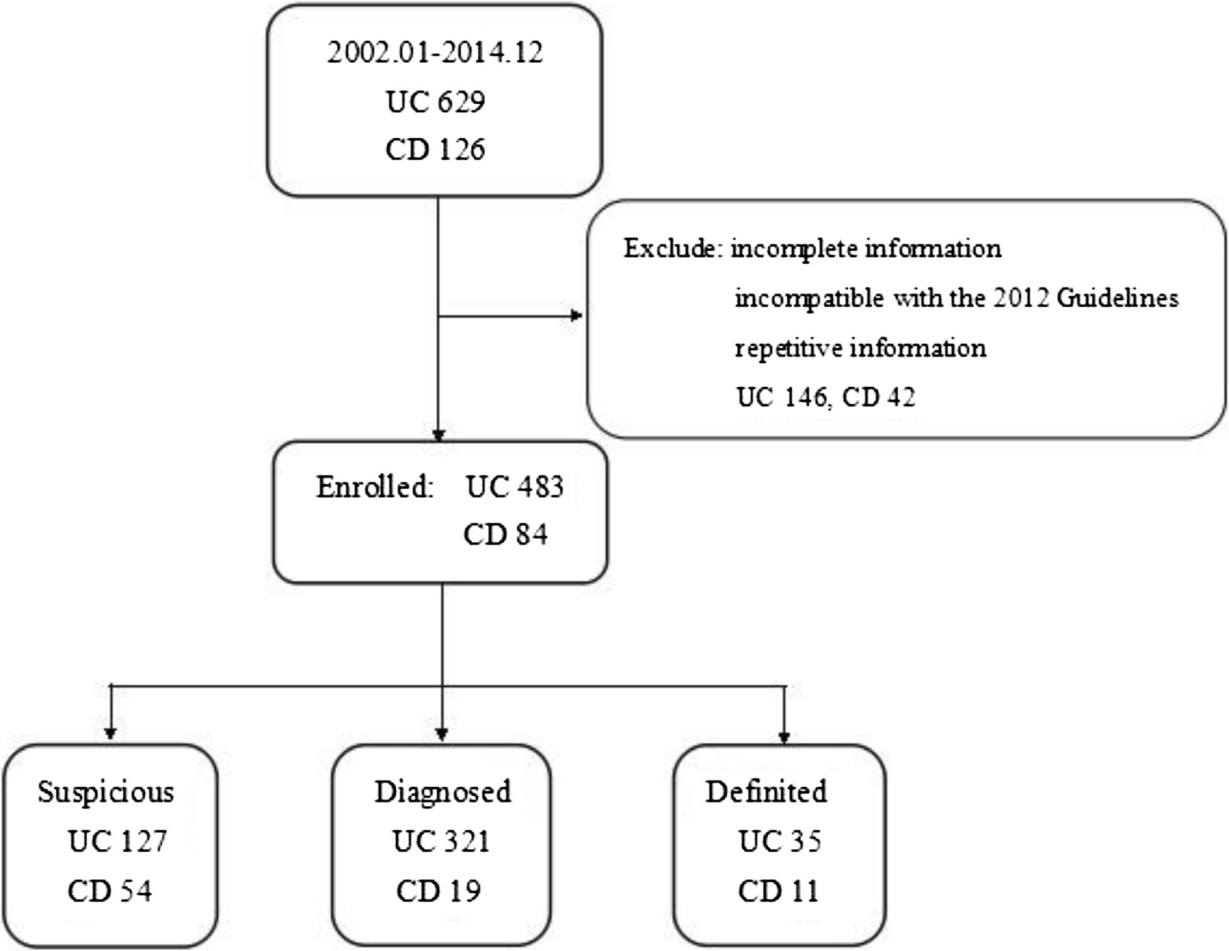
Participant Flow

The aim of this study was to analyze clinical characteristics such as epidemiology, method of diagnosis, and treatment of hospitalized patients with IBD, over a 12-year period at the General Hospital of NingXia Medical University, which is the largest and has a degree of representivity in the region. These data would be a useful observation of the increase in IBD incidence and prevalence in China, and provide valuable information for the proper diagnosis and management of the disease.

## Methods

In this retrospective survey we reviewed the medical records of inpatients with IBD admitted to the General Hospital of NingXia Medical University between January 2002 and December 2014. These high quality records have previously been used as a database for epidemiologic research into diagnoses, treatment, and hospitalizations. Data obtained were checked according to the 2012 guidelines for the diagnosis and treatment of IBD [9]. Moreover, we only recorded the first hospitalization data for repeatedly hospitalized patients (Fig. 1). A standard comprehensive data form was designed to collect general information, for every patient and medical record collected from the Medical Record Database. This information included age, gender, year of diagnosis, clinical manifestations, complications, laboratory tests, treatments (e.g., 5-amino salicylic acid, probiotics, corticosteroids, immunosuppressive agents etc.) and these data were retrospectively analyzed. In addition, we have divided the patients’ occupational physical activity into three degrees. Mild class means they are mainly sedentary and do not walk much around at workplace, for example, desk work, work including assembling of minor parts. Serve class means they have heavy physical work, they carry heavy burdens and carry out physically strenuous work, for example, work including digging and shoveling. Moderate class is the people performing the work whose physical strength is between the mild and severe physical activity. We simultaneously collected data on all gastrointestinal (GI) cases from 2002 to 2014 to calculate the proportion of IBD.

Statistical analysis was performed with SPSS software (version 17.0). All results are expressed as mean (SD) and percentage.

This study was approved by the General Hospital of NingXia Medical University Ethics Committee and is in compliance with the Helsinki Declaration. Access to the health databases is controlled by General Hospital of NingXia Medical University, and was approved by the access committee following review of the application and receipt of ethics approval. All enrolled patients agreed to publish their hospitalized data and have signed the written informed consent.

## Results

### Hospitalization rates

A total of 567 patients, including 483 with UC (85.19 %) and 84 with CD (14.81 %), were examined from 2002 to 2014. The number of GI inpatients admitted generally increased over the study period and an important turning point is seen for 2008 (Table 1). The proportion of GI patients with IBD also increased year on year, with a highest proportion 5.16 %, admitted in 2007. As shown in Table 1, the number (proportion) of IBD patients hospitalized with UC increased from 15 (1.96 %) in 2002 to 59 (3.27 %) in 2014, and with CD from 0 in 2002 to 14 (0.78 %) in 2014. Although the proportion of IBD patients hospitalized for CD is increasing, the vast majority of patients admitted with IBD had UC.

**Table 1:** Proportion of hospitalized patients with IBD of all gastrointestinal patients in Ningxia hospital from 2002–2014

Year	GI Patients (n)	IBD (n/%)	UC (n/%)	CD (n/%)
2002	766	15 (1.96)	15 (1.96)	0
2003	736	16 (2.17)	15 (2.04)	1 (0.13)
2004	900	45 (5.00)	40 (4.44)	5 (0.56)
2005	927	28 (3.02)	21 (2.27)	7 (0.76)
2006	934	32 (3.43)	29 (3.10)	3 (0.33)
2007	872	45 (5.16)	38 (4.36)	7 (0.80)
2008	1246	44 (3.53)	41 (3.29)	3 (0.24)
2009	1334	40 (3.00)	36 (2.70)	4 (0.30)
2010	1629	45 (2.76)	38 (2.33)	7 (0.43)
2011	1642	54 (3.29)	45 (2.74)	9 (0.55)
2012	1768	51 (2.88)	44 (2.49)	7 (0.39)
2013	1762	76 (4.31)	60 (3.41)	16 (0.90)
2014	1803	73 (4.05)	59 (3.27)	14 (0.78)

**Table 2:** Baseline data of 483 patients with ulcerative colitis

	UC
Gender	
Male/Female	266/218 (1.22:1)
Environment	
Rural, n (%)	209 (43.27)
Urban, n (%)	274 (56.73)
Occupational physical activity	
Mild, n (%)	281 (58.18)
Moderate, n (%)	95 (19.67)
Serve, n (%)	107 (22.15)
Age, years, mean ± SD	49.06 ± 14.92
Age at diagnosis, years, mean ± SD	45.98 ± 15.09
Age at diagnosis according to Montreal classification, n (%)	
<17 years	4 (0.83)
17-40 years	129 (26.71)
>40 years	350 (72.46)
Localization of disease according to Montreal classification, n (%)	
E1 Rectum	90 (18.63)
E2 Left colon	160 (33.13)
E3 Extensive colon	233 (48.24)
Severity of intestinal inflammation, n (%)	
Mild	154 (31.88)
Moderate	180 (37.27)
Severe	149 (30.85)
Mayo Score, n (%)	
≦2 and single event ≦1 Clinical remission	179 (37.06)
3-5 Mild activity	11 (2.28)
6-10 Moderate activity	136 (28.16)
11-12 Severe activity	157 (32.50)

**Table 3:** Baseline data of 84 patients with Crohn’s disease

	CD
Gender	
Male/Female	55/29 (1.90/1)
Environment	
Rural, n (%)	31 (36.90)
Urban, n (%)	53 (63.10)
Occupational physical activity	
Mild, n (%)	38 (45.24)
Moderate, n (%)	26 (30.95)
Serve, n (%)	20 (23.81)
Age, years, mean ± SD	44.84 ± 14.67
Age at diagnosis, years, mean ± SD	42.68 ± 17.72
Age at diagnosis according to Montreal classification, n (%)	
A1 ≦16 years	6 (7.15)
A2 17-40 years	29 (34.52)
A3 > 40 years	49 (58.33)
Localization of disease, n (%)	
L1 Ileum	25 (29.76)
L2 Colon	39 (46.43)
L3 Ileocolon	7 (8.33)
L4 Upper gastrointestinal	13 (15.48)
Illness behavior, n (%)	
B1 Non-narrow non-penetrating	60 (71.43)
B2 Stricture	19 (22.62)
B3 Penetrate	5 (5.95)
CDAI, n (%)	
≦4 Remission	45 (53.57)
5-8 Moderate activity	25 (29.76)
≧9 Severe activity	14 (16.67)

**Table 4:** Diagnostic basis

	UC	CD
	n = 483	n = 84
A + D	19 (3.93)	2 (2.38)
A + B	302 (62.53)	17 (20.24)
A + B + C	24 (4.97)	2 (2.38)
A + B + C + D	11 (3.89)	9 (10.71)
Total	356 (73.71)	30 (35.71)

A: Clinical manifestations, B: Colonoscopy, C: Pathology, D: Radiology

### General characteristics

A total of 483 patients with UC were studied, with a male: female ratio of 1.22:1. The majority of these (n = 274; 56.73 %) were living in urban areas, the remainder in rural areas. The most common occupation type was manual work (n = 281; 58.18 %), followed by intermediate work (n = 107; 22.15 %) and mental work (n = 95; 19.67 %). Age ranged from 13 to 88 years, with mean age 49.06 years (SD: 14.92), with no difference in mean age between males and females. According to Montreal classification for age, the majority of patients were >40 years (72.46 %). For disease localization, pancolitis accounted for 233 (48.24 %) of UC inpatients (33.13 % left colon, 18.63 % rectum). Interestingly, the majority of UC patients had moderate luminal inflammation (n = 180; 37.27 %), followed by mild (n = 154; 31.88 %) and severe (n = 149; 30.85 %). Their situation at discharge was assessed based on Mayo Score which include four aspects: defecation frequency, hematochezia, endoscopic and physicians overall assessment, and revealed that after treatment the majority scored ≦2 or single event ≦1. This suggests that on discharge 179 (37.06 %) were in clinical remission, 157 (32.50 %) had mild disease activity and 136 (28.16 %) had moderate disease activity (Table 2).

Of the 84 patients with CD, 55 (65.48 %) were male and 29 (34.52 %) female, giving a male: female ratio of 1.90:1. For environment and occupation a similar tendency to UC was observed for CD patients, with the majority living in urban areas (n = 53; 63.1 %) and engaged in manual labor (n = 31; 36.90 %). However mean age was younger for CD (CD: 44.84 years vs. UC: 49.06). Most frequent site of inflammation was colon (n = 39; 46.43 %), followed by ileum (n = 25; 29.76 %) and upper gastrointestinal (n = 13; 15.48 %), and ileocolon the least often seen (n = 7; 8.33 %). Of the CD patients, 22.62 % had stenosis and 5.95 % had penetrating disease. According to the CDAI [10], 45 (53.57 %) cases were in remission, 25 (29.76 %) had moderate disease activity and 14 (16.67 %) had severe disease activity, and because of their disease condition it was indistinguishable from UC (Table 3).

### Diagnostic methods

A comprehensive diagnostic system, which included clinical manifestation, endoscopy and pathology, was most widely used in the diagnosis of UC (n = 356; 73.71 %); however comprehensive assessment was less commonly carried out for CD (n = 2; 2.38 %), with the diagnosis made on clinical manifestations combined with colonoscopy for the majority of CD patients (n = 17; 20.24 %). It was difficult to establish the value of radiology in UC and CD (Table 4).

### Treatment

The treatment of CD in hospital was relatively basic, with most patients treated with 5-amino salicylic acid and systemic steroid; the treatment of ulcerative colitis was more diverse. Table 5 indicates that more than half (55.69 %) of UC patients were prescribed 5-amino salicylic acid combined with intravenous steroids to control symptoms, followed by SASP/5ASA alone (n = 156; 32.30 %). Among three adjuvant therapies topical treatment was most common (n = 254; 52.59 %) followed by antibiotics (n = 231; 47.83 %). Treatment decisions depended on disease stage and SASP/5ASA only was used mostly for inpatients with mild disease severity (56.49 %) gradually decreasing with disease exacerbation, and only used in 10.07 % of severe cases. Overall, intravenous steroid treatment was more common than oral, and with aggravation of disease its use increased concurrently. Although a similar tendency for immunosuppressive therapy was seen, only seven patients received it, accounting for only 4.7 % of the 149 severely affected patients.

**Table 5:** Treatment and disease severity of 483 patients with ulcerative colitis

	Mild	Moderate	Severe	Total
	n = 154	n = 180	n = 149	n = 483
Antibiotic	52 (33.77)	85 (47.22)	94 (63.09)	231 (47.83)
Topical treatment	36 (23.38)	111 (61.67)	107 (71.81)	254 (52.59)
Chinese medicine (oral/topical)	16 (10.39)	20 (11.11)	35 (23.49)	71 (14.70)
A	87 (56.49)	54 (30.00)	15 (10.07)	156 (32.30)
A + B	16 (10.39)	21 (11.67)	25 (16.78)	52 (10.77)
A + C	47 (30.52)	105 (58.33)	117 (78.52)	269 (55.69)
A + D	0	2 (1.11)	7 (4.70)	9 (1.86)

A: SASP/5ASA, B: Oral steroid, C: Intravenous steroid, D: Immunosuppressive agent

In our study, surgery was performed in 10 UC patients (2.07 %) and 9 CD patients(10.71 %). Of the 10 UC cases, 9 cases were with colonic pseudo-polyps, 1 was with lower gastrointestinal bleeding. Of the 9 CD patients, 3 cases were with anal fistula, 2 cases were with intestinal obstruction, 2 cases were with acute ileal perforation, and 1 case was with rectal fistula bladder, and 1 was with colonic stenosis.

## Discussion

Most recent research has reported an obvious trend of increasing IBD incidence in developing countries; however trends in IBD in North West China have not been subject to investigation and this was the first retrospective study of IBD epidemiology, diagnosis, and treatment in NingXia area. Our study revealed an upward trend in incidence of gastrointestinal patients, with the number of cases of IBD increasing 2.07-fold from 2002 to 2014. Cases of UC increased 1.67-fold and CD increased 5.57-fold in this time period, differs from the reported 2.11-fold UC and 2.78-fold CD over the study years [11]. This difference may be due to the smaller population quota in North West China, limited diagnostic facilities or a relatively slow pace of life leading to a lower incidence of IBD [12]. In Western countries incidence of UC is slowing decreasing and stabilizing, while CD is increasing [13]. However in our study both diseases have increasing rates, possibly because the overall development of IBD in China is lagging behind Western countries. The future direction, i.e., a continued increase or stabilization can only be monitored by further long-term observation.

In our study the male/female ratio was 1.22/1 for UC and 1.90/1 for CD, with both diseases showing a predominating male pattern, especially CD. This difference is consistent with other Asian countries [14], but is not seen in Western countries. We also observed that the majority of patients were from urban areas and took serve occupational physical activity, suggesting environment might play a more prominent role in the etiology of IBD than lifestyle. The mean age of IBD patients in the past 12 years has not changed significantly, but CD shows a younger trend (CD: 44.84 years vs. UC: 49.06 years) and this may be associated with increased awareness of the disease and effective diagnosis.

Differing from the 2007 consensus on diagnosis and treatment of inflammatory bowel disease [15], the 2012 version recommended the Montreal classification which categorizes the location of disease in UC into three sites [16]. In our study extensive colon involvement is the most common presentation (48.24 %), which is inconsistent with the left colon presentation most commonly reported in many Western countries [17]. Although the majority of IBD patients in our study presented with mild to moderate disease activity, patients with severe disease activity accounted for a considerable proportion (30.85 %). This may be due to the study population being hospitalized patients, many of whom may have been admitted due to recurrent disease and worsening symptoms.

Our study concurred with the findings of the APDW 2004 Chinese IBD Working Group, that is, less small bowel disease than colonic disease in Chinese patients [18]; in Western countries small bowel disease is more common than colonic disease [19]. This may reflect a real difference between China and Western countries; however it may be related to widely applied colonoscopy, limited application of capsule endoscopy or small intestinal endoscopy, and a lower rate of surgical intervention in China. Freeman [20] reported that disease localized to ileum alone was most often complicated by stricture formation, whereas ileocolic disease was usually complicated by a penetrating complication. Our study made similar findings, with stricture formation noted in 22.62 % of patients and involvement limited to the ileum in 29.76 % of patients, while ileocolic disease comprised 8.33 % and penetrating disease 5.95 %. This may indicate that Crohn's disease is a dynamic process that phenotypically evolves and progresses with time. In our study CD patients had better clinical condition than UC patients; according to CDAI score 53.57 % of CD patients were in remission.

Our study revealed that clinical manifestation combined with colonoscopy is the most common diagnostic method for both UC and CD; however the 2012 consensus reported colonoscopy and biopsy as the main basis for diagnoses [9]. In our study only 4.97 % patients had findings such as crypt abscess, atrophic crypt, Paneth’s cell metaplasia and other specific histology, causing us to query the role of biopsy in IBD diagnosis.

Interestingly, in our study the site of CD was situated more upper gastrointestinal (15.48 %) than ileocolon (8.33 %) which is the least often seen. This may be owing to referring to the 2012 Guidelines, only very few cases (n = 11,13 %) were definited diagnosed, other 23 % were clinically diagnosed and 64 % were just suspected, so the diagnosis rate are too low to get the accurate conclusion. Moreover, it can also make CD difficult to distinguish from UC and other intestinal diseases, leading to some patients being misdiagnosed or diagnosed with suspected CD. Therefore, the new consensus [9] introduces computed tomography enterography (CTE) and magnetic resonance enterography (MRE) and their wide application may provide a new era for CD diagnosis, however, because of the cost, technology and other issues, the application were limited, how to improve the small intestinal diseases in remote areas has become a major difficulty.

General treatment principles for CD consider the site, behavior and activity of disease before decisions are made in consultation with the patient. However, in our study, the selection of treatment merely depended on clinician experience, and treatment was very basic, mainly using 5-aminosalicylic acid alone or combined with steroids (oral/intravenous). The number of CD cases was relatively small with most cases in remission. Fewer cases may cause clinician inattention, with standardized treatment not implemented. We hope our research creates awareness of the increasing hospitalization rate of CD and adverse effects for patients, so that clinicians can mitigate the development of disease, reducing the burden on patients.

In general, the most widely used treatment of UC was SASP/5ASA combined with intravenous steroids, possibly because our subjects were inpatients with poor disease states and simultaneous intravenous administration is a convenient method of treatment. For mildly affected patients, 56.49 % were treated with SASP/5ASA, and the application of steroids gradually increased with the aggravation of disease, consistent with the recommended guidelines [9]. Using Mayo score [21] at discharge to assess the effects of treatment, our data revealed that the majority of cases were in clinical remission, suggesting effective treatment for most. For cases not in remission at discharge, 28.16 % were classified as moderate and 2.28 % severe, suggesting poor control for some patients. Undoubtedly, this is related to extremely limited application of immunosuppressive agents, even in patients with severe disease activity, with only 4.7 % receiving this treatment option.

Despite improved aware of the serious consequences of antibiotic abuse and measures to limit their use in recent years, 47.83 % of patients in our study were treated with antibiotics. This number grew with exacerbation of disease, in some cases due to a genuine need due to cytomegalovirus (CMV) or clostridium (CDI) infection. In other cases, poor control by essential drugs may have resulted in the use of experimental antibiotic therapy. It has been reported that the prevalence of CMV infection in acute severe colitis is between 21 % and 34 % [22, 23], and the prevalence of CDI infection increased from 1.8 % in 2004 to 4.6 % in 2005 [24]. Even though there are multiple diagnostic tests available for detecting infection, there are problems with these tests which remain controversial. Diagnosis and treatment standardization of these infections will possibly mitigate the abuse of antibiotics in IBD.

Chinese medicine played an important role, especially in patients with moderate-severe activity, this is different from the western countries. However, its specific role and efficient components are still unknown and need further study. Relatively the operation rate is low, and CD patients were more frequent (10.71 %, UC 2.07 %), fistula and stenosis were the main reason.

In addition, only 8.86 % UC and 13.09 % CD patients can get a definite diagnosis in our study, 66.46 % UC and 22.62 % CD were clinically diagnosed, 26.29 % UC and 64.29 % CD were just suspicious. Perhaps there are some indeterminate colitis. However, due to the lack of understanding of this type, it was classified as UC or CD based on the physicians’ clinical experience in actual clinical diagnosis. By reviewing the medical records of inpatients with IBD over 12 years and the further learning the guidelines, we hope to increase the awareness of IBD and the understanding of indeterminate colitis in the future clinical practice.

This was a retrospective analysis, and the lack of follow-up information meant we were unable to make a comprehensive evaluation of treatment effectiveness. However, it is evident that the monitoring of IBD patients should be strengthened, with the establishment of a comprehensive follow-up system.

## Conclusions

An increasing trend of admissions for IBD can be observed in our study; there are some differences in clinical features and treatment compared with Western countries, and further research into this is required.
